# Characterization of dabrafenib-induced drug insensitivity via cellular barcoding and collateral sensitivity to second-line therapeutics

**DOI:** 10.1038/s41598-023-50443-3

**Published:** 2024-01-02

**Authors:** Rana Can Baygin, Kubra Celikbas Yilmaz, Ahmet Acar

**Affiliations:** Department of Biological Sciences, Middle East Technical University, Universiteler Mah. Dumlupınar Bulvarı 1, Çankaya, 06800 Ankara, Turkey

**Keywords:** Cancer models, Tumour heterogeneity

## Abstract

Drug insensitivity is arguably one of the biggest challenges in cancer therapeutics. Although effective therapeutic solutions in cancer are limited due to the emergence of drug insensitivity, exploiting evolutionary understanding in this context can provide potential second-line therapeutics sensitizing the drug insensitive populations. Targeted therapeutic agent dabrafenib is used to treat CRC patients with BRAF V600E genotype and insensitivity to dabrafenib is often observed. Understanding underlying clonal architecture of dabrafenib-induced drug insensitivity and identification of potential second-line therapeutics that could sensitize dabrafenib insensitive populations remain to be elucidated. For this purpose, we utilized cellular barcoding technology to decipher dabrafenib-induced clonal evolution in BRAF V600E mutant HT-29 cells. This approach revealed the detection of both pre-existing and de novo barcodes with increased frequencies as a result of dabrafenib insensitivity. Furthermore, our longitudinal monitoring of drug insensitivity based on barcode detection from floating DNA within used medium enabled to identify temporal dynamics of pre-existing and de novo barcodes in relation to dabrafenib insensitivity in HT-29 cells. Moreover, whole-exome sequencing analysis exhibited possible somatic CNVs and SNVs contributing to dabrafenib insensitivity in HT-29 cells. Last, collateral drug sensitivity testing demonstrated oxaliplatin and capecitabine, alone or in combination, as successful second-like therapeutics in inducing collateral sensitivity in dabrafenib-insensitive HT-29 cells. Overall, our findings demonstrate clonal dynamics of dabrafenib-insensitivity in HT-29 cells. In addition, oxaliplatin and capecitabine, alone or in combination, were successful second-line therapeutics in inducing collateral sensitivity in dabrafenib-insensitive HT-29 cells.

## Introduction

Cancer is a complex disease characterized by the formation of malignant cells^1,2^. Malignant cell characteristics are summarized as their growth signal self-sufficiency, ability to evade apoptosis, anti-growth signal insensitivity, angiogenesis, invasion and metastasis, replicative potential, reprogramming energy metabolism, and immune system evasion capacity^3^.

Colorectal cancer (CRC) ranks second with 9.4% mortality and third with 10% of all cancers^4^. Defined by its heterogenous nature in the clinic, CRC has been classified by genomic and transcriptomic characterization using next-generation sequencing approaches^5^. Genomic characterization resulted in defining two major classes namely hypermutated, harboring microsatellite instability (MSI), and non-hypermutated cancers with microsatellite stability (MSS)^6^. Transcriptomic characterization performed by Consensus Molecular Subtypes (CMS) consortium outlined MSI cancers into one category as CMS1 (MSI-immune) and MSS tumors into three categories as CMS2 (canonical), CMS3 (metabolic), and CMS4 (mesenchymal)^6^. The underlying determinants of genomic and transcriptomic tumor heterogeneity have attracted the attention of researchers, particularly in the last decade^7^. Intra-tumor heterogeneity (ITH) is caused by genetic changes as well as epigenetic mechanisms such as chromatin remodeling, DNA methylation, and histone post-translational modifications^8^. Tumor heterogeneity influences the emergence of tumor subclones during tumor progression, which contributes to treatment insensitivity and, ultimately, failure^9^. The ITH is strongly influenced by the selection of pre-existing subclones and the emergence of de novo forms^10^. One of the key determinants of this process is Darwinian evolution, which asserts that the selective pressure of the treatment utilized facilitates the growth and survival of the fittest^11–13^. For example, the presence of therapeutic pressure can alter tumour phenotype to better adapt to different evolutionary speeds managed by macro-evolutionary events such as large chromosomal alterations and hypermutations^12,14^. Selective therapeutic pressure and genomic drivers can lead to therapy failure, often via insensitivity mechanisms^15^.

Dabrafenib is a BRAF V600E inhibitor that interferes with the RAF/MEK pathway^16^. Dabrafenib is a five-membered heterocyclic group with a central ring locking and affinity for binding BRAF; as a result, there is reversible competitive inhibition of potent adenosine triphosphate, which selectively inhibits the BRAF (V600E) kinase domain^17^. Although BRAF inhibitors could inhibit its target, BRAF V600E inhibitors can only inhibit the mutated BRAF protein, which is a monomer, rather than the wild-type BRAF dimer^18^. In RAF-mutated cells, activation of MEK/ERK (which is found downstream of RAS/RAF) induces cell survival and proliferation^19–21^. As a result, negative feedback on MAPK pathway inhibition causes the formation of RAF dimers and insensitivity to RAF inhibitors, which may be the primary cause of drug insensitivity^22^. In CRC, targeting multiple MAPK pathway members can improve the anti-tumour effect of treatment^23,24^.

The genome barcoding technology allows for the tracking of evolutionary lineages as well as drug insensitivity^25,26^. The frequency of selected clones and their fitness effect, as a result of treatment, can be calculated by the cellular barcoding technology^26,27^. Using DNA barcoding technology, previous studies shed light on the molecular determinants of drug insensitivity and the emergence of pre-existing and de novo drug insensitivity^25–29^. For example, we have previously showed the presence of polyclonal drug insensitivity in barcoded non-small cell lung cancer cell line HCC827 by exposing it to gefitinib and trametinib separately in an experimental evolution setting. In this study, pre-existing and de novo drug insensitivity were identified by calculating barcode frequencies in both harvested drug insensitive cells and temporal tracking of clonal evolution from used medium^26^. Understanding clonal evolution under selective drug pressure can help in identification of tumours’ vulnerability to second-line therapy, also known as collateral drug sensitivity^30^. Few studies, including ours, have demonstrated that drug-insensitive cancer cells can become more sensitive to second-line therapies^26,31–33^. As a result, collateral sensitivity approaches that rely on administering a sequence of inhibitors may be advantageous to patients in terms of less toxicity^34^.

In this study, we show dabrafenib-induced drug insensitivity in HT-29 cells consisted of both pre-existing and de novo mechanisms using high-resolution cellular barcoding technology. Barcoding method also enabled longitudinal tracking of dabrafenib insensitivity in HT-29 cells via collection and sequencing of floating barcodes. Also, whole-exome sequencing analysis performed to dissect dabrafenib insensitivity in HT-29 cells revealed the contribution of CNVs and SNVs in replicate A harboring dominant pre-existing barcodes and hence detected variations might be attributed to these pre-existing barcodes. Furthermore, dabrafenib-induced insensitivity in HT-29 cells caused decreased cellular migration, presumably due to the cost of insensitivity. Exploiting the cost of dabrafenib-induced drug insensitivity in HT-29 cells showed increased sensitivity to oxaliplatin and capecitabine alone or their combination as potential collaterally sensitive drugs. Collectively, we have showed clonal evolution of dabrafenib-induced drug insensitivity in HT-29 cells using barcoding technology while also demonstrating collateral drug sensitivity in dabrafenib-insensitive HT-29 cells.

## Results

### Barcoding of HT-29 and determining dabrafenib sensitivity in HT-29 cells

**Figure 1:**
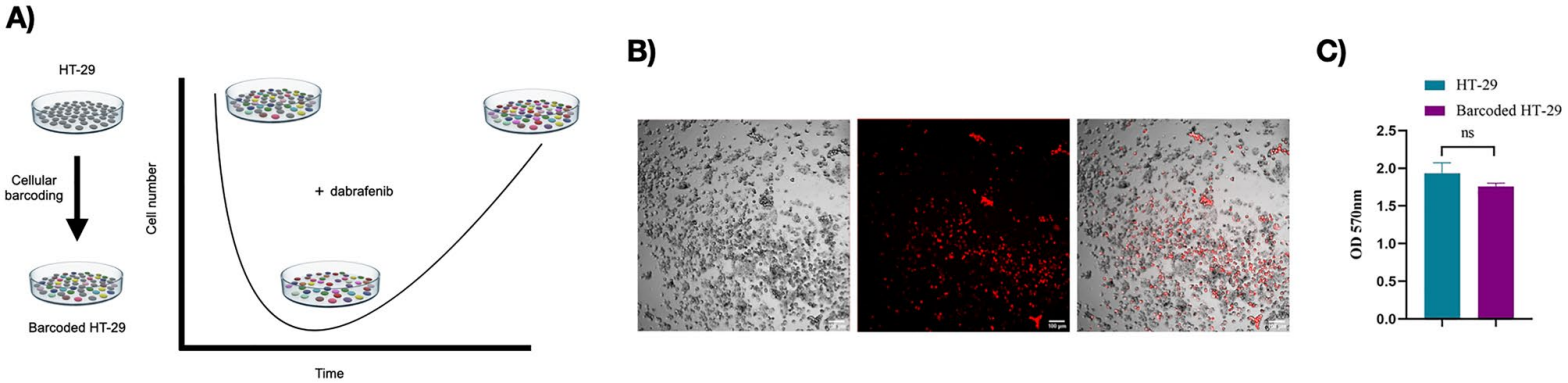
Experimental set-up and barcoding of HT-29 cells. (**A**) Design for barcoding HT-29 cell lines and insensitivity to dabrafenib. (**B**) Microscope images of barcoded HT-29 cells using lentiviral barcode library including red fluorescent protein (RFP). (**C**) Proliferation assay performed for non-barcoded and barcoded HT-29 cells. Error bars represent SEM. Two-way ANOVA was used as a statistical test. *ns* non-significant. P > 0.05.

To examine the clonal dynamics of dabrafenib insensitivity in HT-29 cell line, cellular barcoding technology was utilized (Fig. 1A). Multiplicity of infection (M.O.I) 0.05 was aimed to achieve for integration of unique barcodes in initial HT-29 cell line. Since lentiviral cassette harboring individual barcodes also had red fluorescent protein (RFP), this was confirmed by checking the expression of RFP expression using fluorescent microscope (Fig. 1B). To assess potential effect of the integration of barcodes in HT-29 cell line, cell proliferation assay was performed in non-barcoded and barcoded HT-29 cell lines. This assay demonstrated no significant proliferation difference in a barcoded HT-29 cell line in comparison to a non-barcoded cell line (Fig. 1C). As a result, HT-29 cell line was modified with barcode integration, which had no effect on cellular proliferation.

### Establishing dabrafenib-insensitive derivatives of barcoded HT-29 cell line

**Figure 2:**
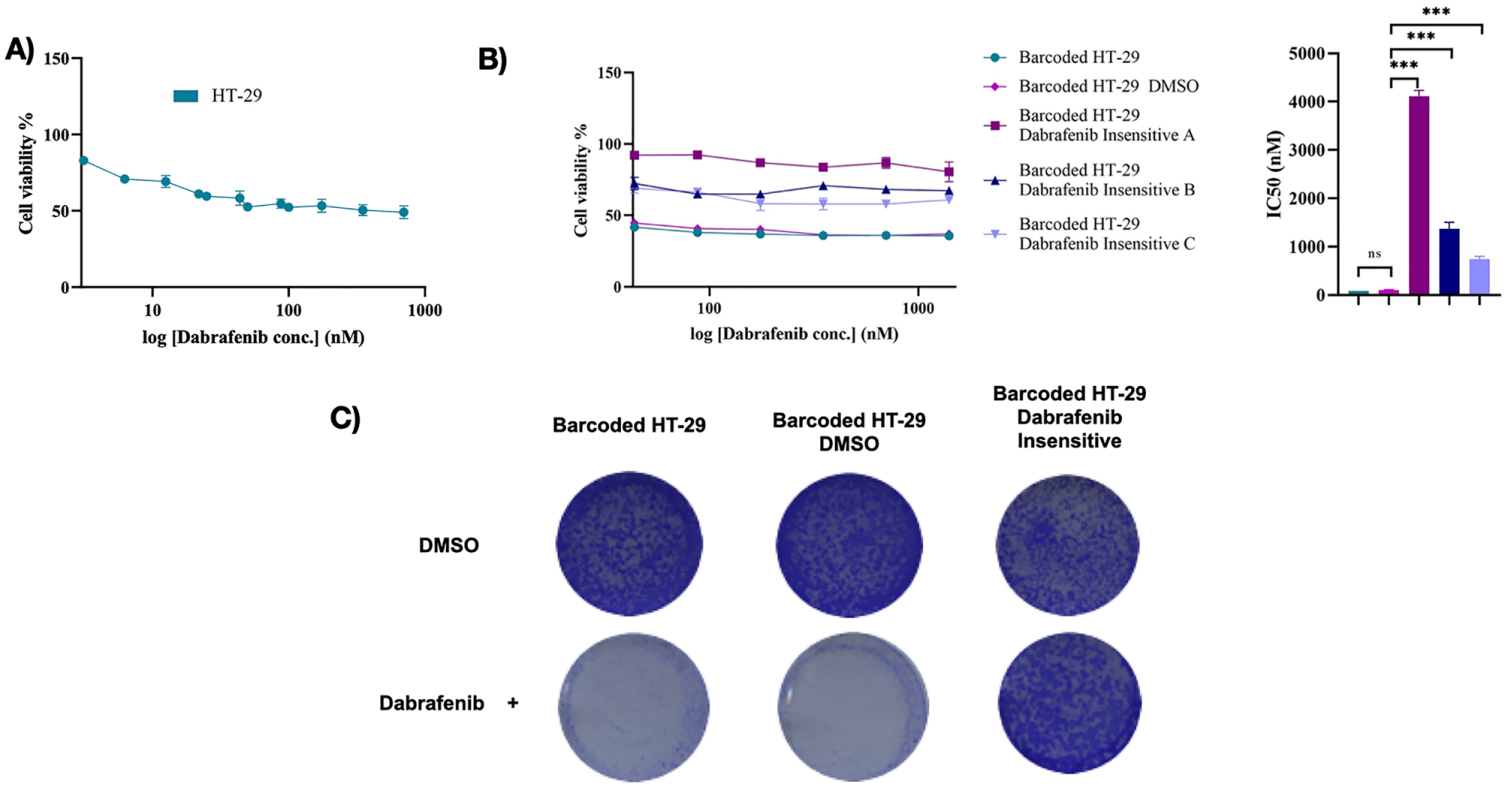
Generation of dabrafenib-insensitive HT-29 cell lines. (**A**) MTT cell viability assay in HT-29 cell lines for dabrafenib dose–response curve. (**B**) MTT cell viability assay for dabrafenib-insensitive HT-29 replicates A, B, and C, as well as the DMSO control, initial population, and non-barcoded parental cell lines. (**C**) Colony formation assay dabrafenib-insensitive HT-29 replicate A, DMSO control, initial population under DMSO and 199.6 nM dabrafenib treatment. Error bars represent SEM. One-way ANOVA test was used to determine statistical significance. ***P < 0.001.

As the drug treatment invariably generates drug insensitivity in cancer cells and quantitative measurement of drug insensitivity have not been previously elucidated in this context, we aimed to approach this problem using cellular barcoding technology. HT-29 cell line carrying BRAF V600E mutation, a known target for dabrafenib, were exposed to dabrafenib to establish its drug insensitive derivative. To establish dabrafenib-insensitive derivatives of HT-29 cell line, we set up an experiment whereby barcoded HT-29 cell lines were exposed to dabrafenib for a duration of 3 months until the drug insensitive derivatives were established. Dabrafenib treatment was performed in three parallel replicates, namely replicate A, B and C, to monitor shared and non-shared barcode frequency measurement based on previous method^26^. After establishment of barcoded HT-29 cell population, dabrafenib sensitivity in these cells were determined. For this purpose, MTT cell viability assay was performed and IC50 concentration of dabrafenib was found as 99.8 nM in barcoded HT-29 cell line (Fig. 2A). Since initial attempts to establish dabrafenib insensitivity in HT-29 cell lines using 1XIC50 (99.8 nM) concentration was not successful, we exposed HT-29 cells to 2XIC50 (199.6 nM) dabrafenib concentration. Following the exposure of barcoded HT-29 cells to 199.6 nM dabrafenib concentration for the duration of 3 months in 3 parallel HT-29 replicates, MTT viability assay was performed. Cell viability assay showed IC50 values of barcoded HT-29 replicate A, B, and C as 4235 nM, 1229 nM, 677 nM, respectively and increased insensitivity to dabrafenib in replicate A (51.04-fold), B (14.80-fold) and C (8.16-fold) in comparison to barcoded DMSO treated HT-29 cells while DMSO treated and non-treated groups exhibited similar dabrafenib sensitivity (Fig. 2B, Supplementary Table 1). Furthermore, we validated dabrafenib insensitivity in replicate A, with highest insensitivity observed through MTT analysis, using colony formation assay. Crystal Violet (CV) staining showed elevated colony formation in dabrafenib insensitive HT-29 cells in comparison to DMSO and initial HT-29 cells in the presence of dabrafenib treatment while the same group of cells under the treatment of DMSO exhibited similar degree of colony formation capacity, indicating the insensitivity of dabrafenib-insensitive HT-29 replicate A to dabrafenib treatment (Fig. 2C). Together, we determined the sensitivity of the HT-29 cell line to dabrafenib and established dabrafenib insensitive barcoded HT-29 cell line replicates A, B and C, among these replicates, the replicate A showed the highest insensitivity to dabrafenib.

### Quantitative measurement of dabrafenib insensitivity in barcoded HT-29 cell lines

**Figure 3:**
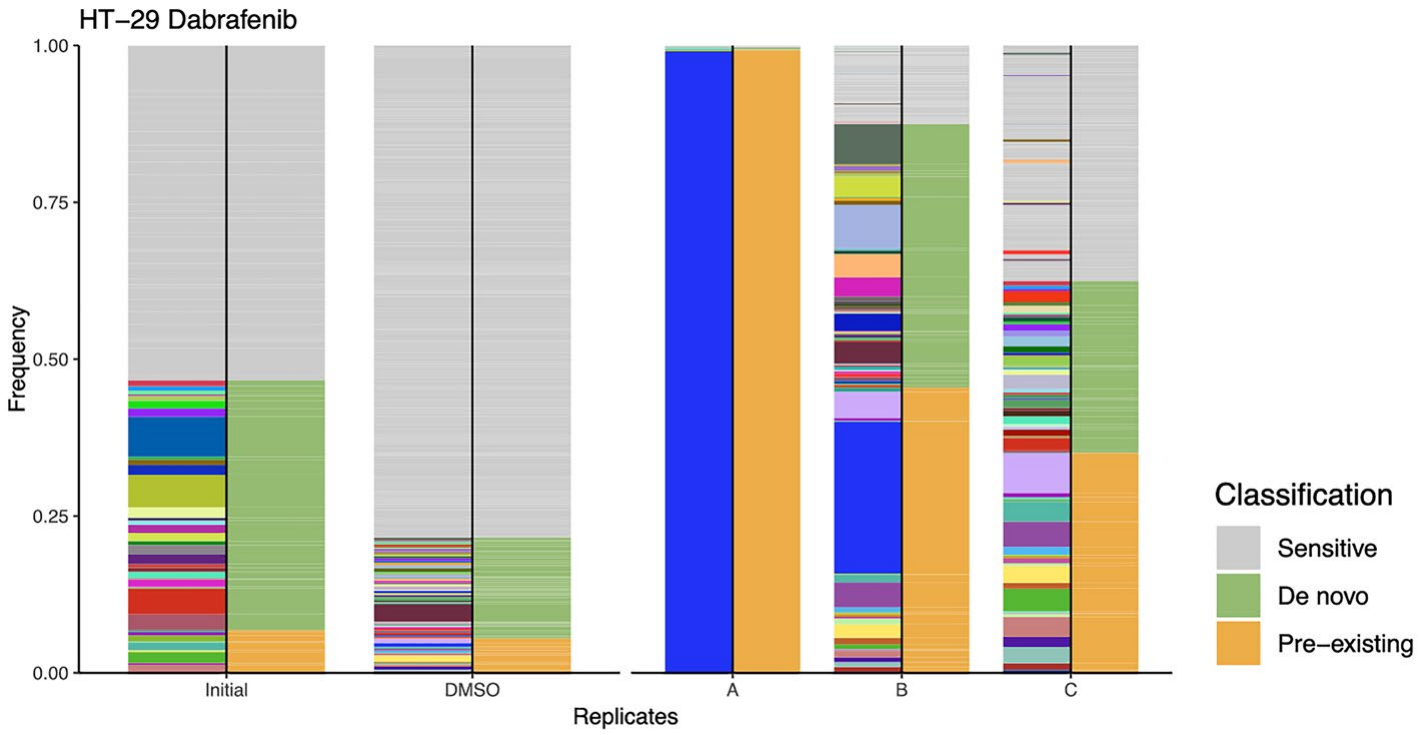
Barcode frequency distributions in dabrafenib-insensitive HT-29 cell lines. The measurements of pre-existing barcode frequencies in initial, DMSO and dabrafenib-insensitive replicate A, B and C in HT-29 cells are shown. The barcode phenotypes are shown with different colors (amber: pre-existing, olive green: de novo, silver grey: sensitive) on the right-hand side of bars. Positive growth rate barcodes were classified as pre-existing or de novo.

To investigate evolutionary drug-induced clonal selection, the barcode frequency distributions were measured in dabrafenib-insensitive HT-29 replicates, initial and control DMSO HT-29 cell lines. To do so, genomic DNA (gDNA) isolation from the cell lines were performed which was then followed by barcode amplification and sequencing using next generation sequencing (NGS). To determine pre-existing drug insensitivity, we tracked same barcodes which exhibited positive growth rate in all of the replicates under same drug relative to control DMSO group (please see methods for details). Barcodes with positive growth rate in individual replicates were determined as potentially de novo, while barcode frequencies demonstrated negative growth rate called as sensitive. For dabrafenib insensitive HT-29 cell lines, there were 4 unique pre-existing barcodes in replicate A, 25 in replicate B, and 23 in replicate C (Supplementary Table 2). Furthermore, de novo barcode identification in dabrafenib-insensitive HT-29 cell lines revealed as 8 in replicate A, 42 in replicate B, and 36 in replicate C (Supplementary Table 2). The pre-existing barcode frequencies (amber color) positively selected under dabrafenib with positive growth rate with respect to control DMSO group was found as 99.3% in replicate A, 45.47% in replicate B, and 35.07% in replicate C (Fig. 3). Moreover, potentially de novo barcode frequencies (olive green color) were detected as 0.37% in replicate A, 41.99% in replicate B, and 27.37% in replicate C (Fig. 3). Last, sensitive barcodes (silver gray color) with negative growth rate was identified as 0.32% in in replicate A, 12.54% in replicate B and 37.56% in replicate C within dabrafenib-insensitive HT-29 cell lines (Fig. 3). These findings indicated that dabrafenib insensitivity in HT-29 cells were governed by both pre-existing and de novo drug insensitivity, suggesting polyclonal drug insensitivity in our experimental setting.

### Temporal tracking of dabrafenib-insensitivity in HT-29 cells using cellular barcoding technology

**Figure 4:**
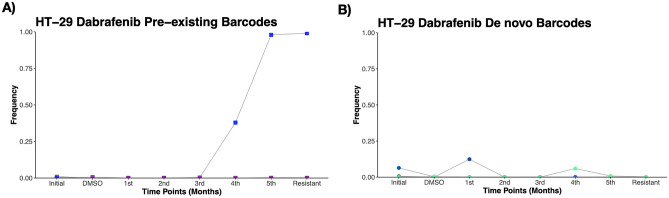
Temporal analysis of clonal evolution in drug-insensitive cells. (**A**) Detection and measurement of the frequencies of pre-existing barcodes from used medium of dabrafenib-insensitive HT-29 cell were shown. (**B**) Identification and measurement of the frequencies de novo barcodes in floating medium of dabrafenib-insensitive HT-29 cells were demonstrated. The barcode frequency in the last data point nearly matches the barcode frequency in the harvested (insensitive) sample point. Each barcode is represented by a different color.

Next, we aimed to measure dabrafenib insensitivity temporally in HT-29 cells. To achieve this, used medium of barcoded HT-29 cells harboring shed DNA particles with barcodes were harvested and gDNAs were isolated before the barcode sequencing and bioinformatics analysis. Of note, majority of shed DNA carrying barcodes are expected to be released from drug sensitive cells, however minority population attached to the tissue culture plates could still die and release their barcode into the medium due to their detachment from the plate surface, representing drug insensitive population. For this approach, replicate A of dabrafenib insensitive HT-29 cell line, with highest dabrafenib insensitivity, was used. Longitudinal barcode analysis of dabrafenib insensitivity in HT-29 replicate A showed that a total of 3 unique pre-existing barcodes were detected with increased frequency at each monthly intervals and closely matched with the final barcode frequency determined from harvested cell population (Fig. 4A). Furthermore, de novo barcodes tracked from used medium with a total number of 2 showed positive growth rate and their frequencies nearly matched with the final harvested barcode frequencies (Fig. 4B). Moreover, our investigation on barcodes with negative growth rate also revealed the detection of sensitive barcodes from used medium in dabrafenib-insensitive HT-29 cells during the course of 3 months (Supplementary Fig. 1A). Hence, non-destructive temporal tracking of dabrafenib insensitivity allowed us to determine ongoing dabrafenib insensitivity induced selection in HT-29 cell line, quantitatively.

### Genomic characterization of dabrafenib-induced insensitivity in HT-29 cells

**Figure 5:**
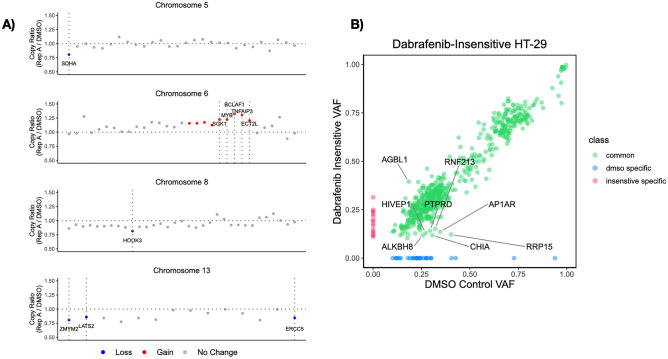
Whole-exome sequencing analysis identified somatic genetic alterations in dabrafenib-insensitive HT-29 cells. (**A**) Copy number variation (CNV) analysis of dabrafenib-insensitive HT-29 replicate A relative to control DMSO HT-29 cells exhibited a gain in *ECT2L*, *MYB*, *SGK1*, *TNFAIP3*, *ROS1* genes and a loss in *LATS2*, *ZMYM2*, *ERCC5*, *HOOK3*, *SDHA* genes. (**B**) The depletion of Variant Allele Frequency (VAF) in dabrafenib-insensitive HT-29 replicates in comparison to control DMSO HT-29 cells revealed somatic SNVs in *CHIA*, *AP1AR*, *HIVEP1*, *PTPRD*, *ALKBH8*, and *RNF213* genes and the enrichment of VAF-showed a somatic SNV in *AGBL1* gene.

We next asked whether dabrafenib-induced insensitivity has caused genomic changes in dabrafenib-insensitive HT-29 cells. To do so, we performed whole-exome sequencing (WES) analysis in DMSO-treated HT-29 and dabrafenib-insensitive HT-29 replicate A with an ultimate aim to compare enriched genetic alterations in the insensitive cells. We initially performed copy number variation (CNV) analysis and found that focal amplifications in *ECT2L*, *MYB*, *SGK1*, *TNFAIP3*, *ROS1* genes and deletions in *LATS2*, *ZMYM2*, *ERCC5*, *HOOK3*, *SDHA* genes in dabrafenib-insensitive HT-29 in comparison to DMSO-treated HT-29 cells (Fig. 5A**)**. The *C-MYB* has been previously reported to facilitate malignant progression of CRC through epithelial-to-mesenchymal transition and proposed as a potential prognostic biomarker^35^. In addition, upregulation of *SGK1* was previously reported in CRC samples in comparison to nearby normal tissue as well as promoting cellular proliferation, migration, and the inhibition of apoptosis^36^. Moreover, *SGK1*, downstream of YAP1 transcriptional activator, has been reported to activate ERK1/2 signalling and promote BRAF V600E insensitivity in metastatic CRC cells^37^. Lastly, downregulation of *LATS2* was reported to be linked to progression and metastasis of CRC^38^. Moreover, decreased expression of *LATS2* was associated with TNM stage and significant independent prognostic factor in CRC patients^39^.

Next, we sought to identify possible single-nucleotide variations (SNVs) in dabrafenib- insensitive HT-29 replicate A in comparison to DMSO-treated HT-29 cells. Our analysis indicated the depletion of a total 7 SNVs and enrichment of a total 1 SNV in dabrafenib-insensitive HT-29 replicate A in comparison to DMSO-treated HT-29 cells (Fig. 5B**)**. These were *AGBL1* (ENST00000441037.7): c.439C>G p.L147V, *CHIA* (ENST00000369740.6): c.817C>A p.L273M, *RRP15* (ENST00000366932.4): c.435G>T p.M145I, *AP1AR* (ENST00000274000.10): c.281_282insGGT p.94_95insV, *HIVEP1* (ENST00000379388.7): c.4730C>T p.P1577L, *PTPRD* (ENST00000381196.8): c.1378A>T p.T460S, *ALKBH8* (ENST00000428149.6): c.110C>A p.T37K, *RNF213* (ENST00000508628.6): c.1474C>T p.H492Y. Among the mutations we detected, a previous study reported the fusion of *ABGL1-NTRK3* gene in a sigmoid colon cancer patient with pulmonary metastasis, underlying mechanisms are yet to be elucidated^40^. Furthermore, *RRP15* was reported to be upregulated in primary CRC samples and there was a correlation of the higher expression of *RRP15* with TNM stage^41^. In addition, another study demonstrated that elevated expression levels of *RRP15* in colon cancer patient samples in comparison to normal tissue and exhibited poorer overall survival and disease-free survival in these patients^42^. Taken together, genomic characterization of DMSO-treated HT-29 and dabrafenib-insensitive HT-29 replicate A cells suggested possible pre-existing mechanisms of insensitivity mediated by CNVs and SNVs in certain genes facilitating the drug insensitivity considering pre-existing barcode govern the 99.3% of the replicate A population.

### Effect of dabrafenib insensitivity on growth rate and migration of barcoded HT-29 cell line

**Figure 6:**
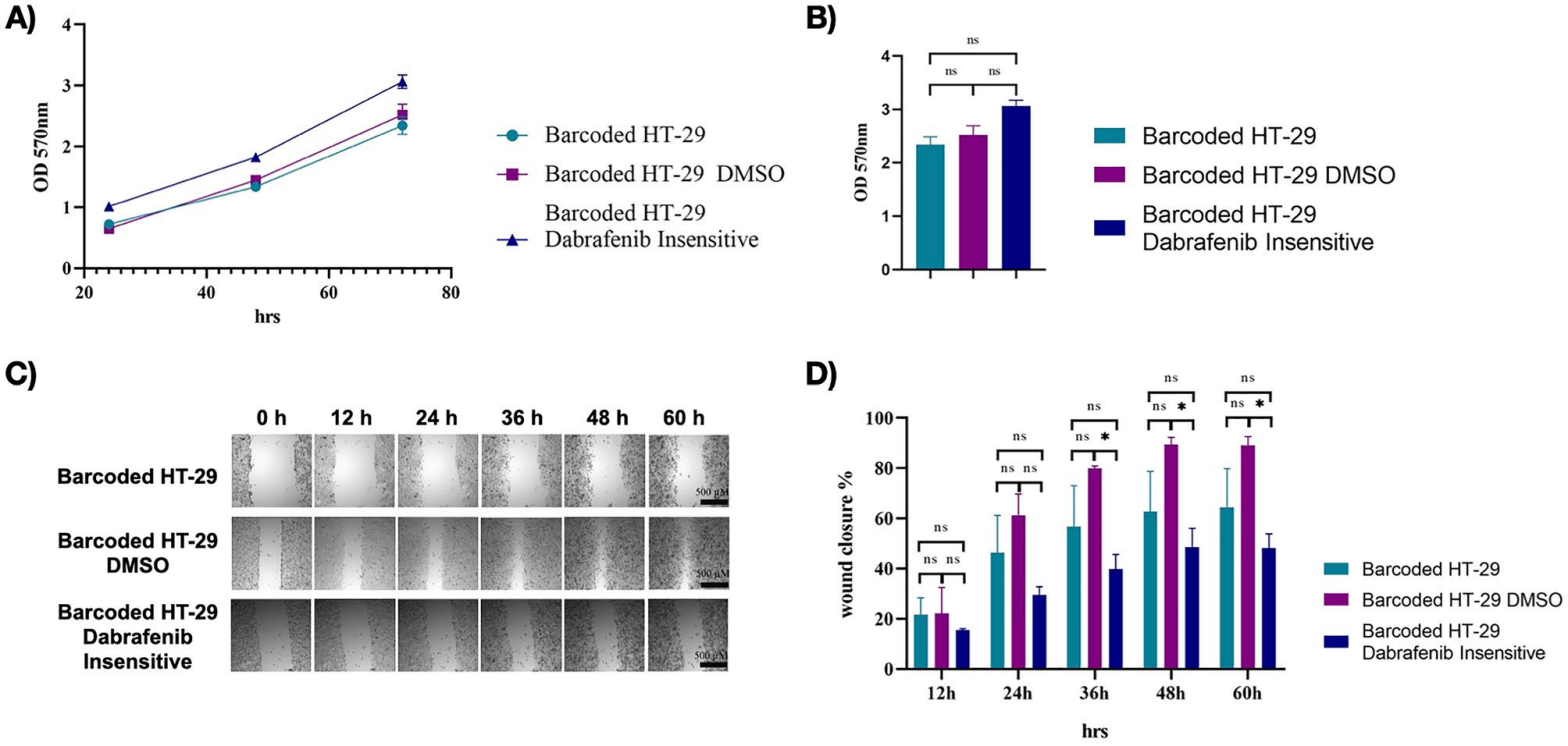
Characterization of dabrafenib-insensitive HT-29 cells. (**A**) Growth rate measurements were carried out for dabrafenib-insensitive HT-29 replicate A, DMSO, and initial HT-29 cells every 24 h. (**B**) Identification of proliferation rate differences in dabrafenib-insensitive HT-29 replicate A, DMSO, and initial HT-29 cells. (**C**) Microscope images of scratch assay obtained at 24 h, 36 h, 48 h, and 60 h in dabrafenib-insensitive HT-29 replicate A and DMSO control HT-29 cell lines. (**D**) A scratch assay based on the percentage for closure of the wound area the was performed to determine the migration rates of dabrafenib-insensitive HT-29 replicate A and DMSO control HT-29 cell lines at 24 h, 36 h, 48 h, and 60 h intervals. SEM was represented by the error bars. Two-way ANOVA test was used to determine statistical significance. *ns* non-significant. *P < 0.05.

We next sought to identify whether dabrafenib insensitivity in HT-29 cells might have any effect on growth rate, and cellular migration. Insensitivity to a drug can come with a cost of sensitivity to second-line drug(s) which has been proposed as evolutionary trade-offs and studied in adaptive therapy^26,43–45^. To assess the growth rates in barcoded dabrafenib insensitive, DMSO control and initial HT-29 populations, cells were fixed, and crystal violet staining was performed at 24 h, 48 h and 72 h. This was then followed by dissolving of the staining and calorimetric quantification. As a result, we observed was no change in growth rates at time points 24 h, 48 h and 72 h in dabrafenib insensitive HT-29 cells relative DMSO control group, indicating that dabrafenib insensitivity in HT-29 cells had no effect on growth rate (Fig. 6A). In addition, no difference in cell proliferation rates in dabrafenib insensitive HT-29 cells relative to control DMSO and initial HT-29 populations at 72 h were visualized (Fig. 6B). To further investigate whether dabrafenib insensitivity might cause a change in migration abilities of HT-29 cells, barcoded initial, control DMSO and dabrafenib insensitive HT-29 cells were examined via scratch assay and resulting microscope images were obtained at different time points (12 h, 24 h, 36 h, 48 h and 60 h) (Fig. 6C). In this assay, percentage of closed area (wound closure %) in sample groups were calculated at 12 h, 24 h, 36 h, 48 h and 60 h. This analysis indicated a 28% decrease in wound closure in dabrafenib-insensitive HT-29 cells relative to DMSO control while there was no statistically significant change in comparison to initial HT-29 cells, suggesting a cost of dabrafenib insensitivity in HT-29 resulting in decreased cellular migration (Fig. 6D). Collectively, despite no effect of dabrafenib insensitivity in growth rate in dabrafenib-insensitive HT-29 cells, the migratory phenotypes of dabrafenib insensitive HT-29 cells were downregulated in comparison to control DMSO HT-29 cell populations.

### Capecitabine and oxaliplatin as a second line therapy increases vulnerabilities in dabrafenib-insensitive HT-29 cells

**Figure 7:**
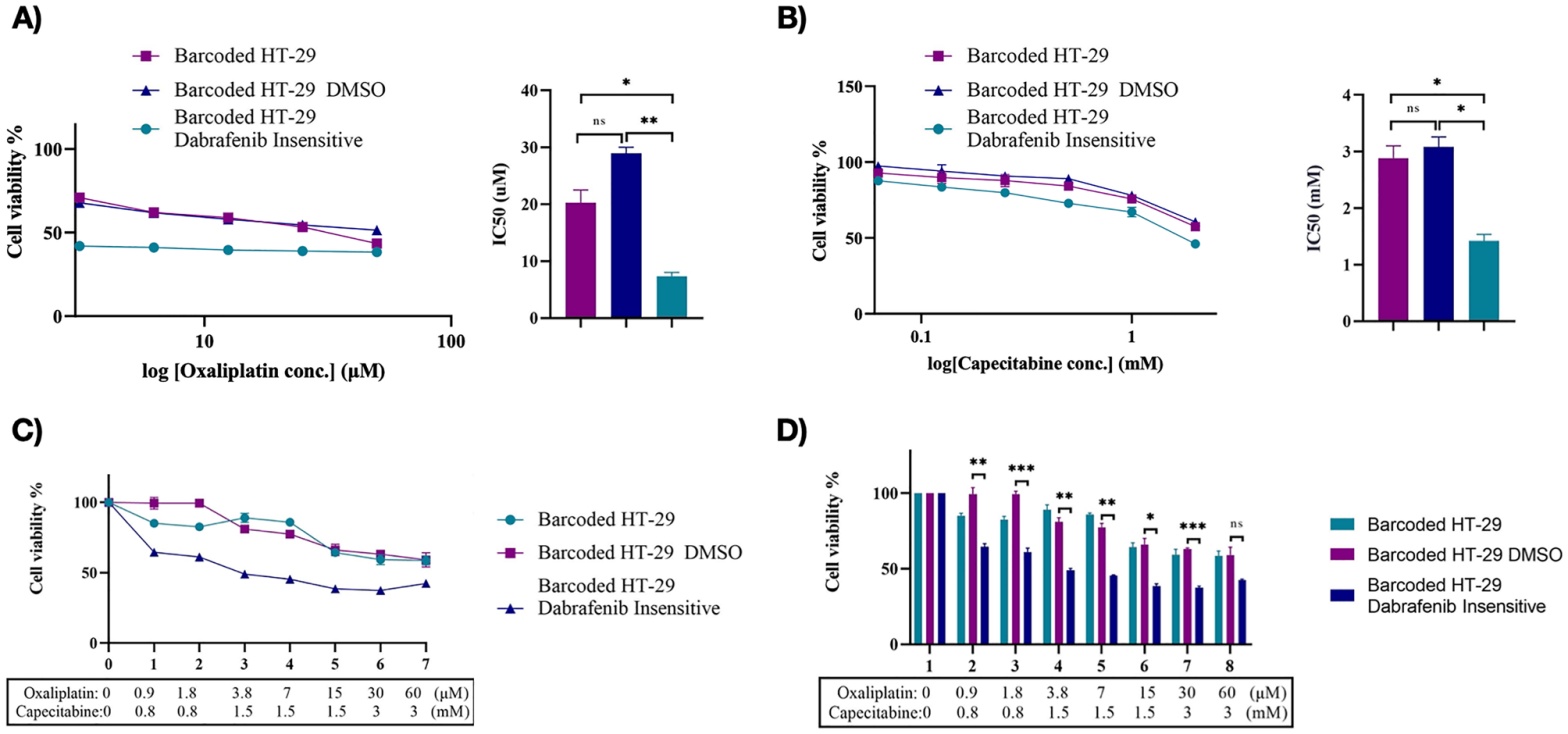
Identification of collateral drug sensitivity in dabrafenib-insensitive HT-29 cells. (**A**) Dose–response curve analysis revealed collateral sensitivity to oxaliplatin in dabrafenib-insensitive HT-29 replicate A. (**B**) Analysis of the dose–response curve in dabrafenib-insensitive HT-29 replicate A revealed collateral sensitivity to capecitabine. (**C**) Dose–response curve analysis of oxaliplatin and capecitabine combination showed collateral sensitivity in dabrafenib-insensitive HT-29 cells. (**D**) Bar chart presentation of oxaliplatin and capecitabine combination sensitivity was measured in dabrafenib-insensitive HT-29 cells and showed increased sensitivity in comparison to DMSO control HT-29 cells. SEM was represented by the error bars. One-way ANOVA test was used to determine statistical significance. *ns* non-significant. *P < 0.05, **P < 0.01, ***P < 0.001.

The evolutionary therapy has been proposed as a cost of insensitivity sensitizing the insensitive population to a second line therapy^30,44,46^. This phenomenon was termed as collateral drug sensitivity has been shown to be effective in bacteria^47,48^, malaria^49^ and cancer^32,50^, including in our recent study^26^. In this context, evolutionary steering induced by dabrafenib insensitivity in HT-29 cells can cause a change in evolutionary dynamics in these cell lines. Since capecitabine and oxaliplatin were administrated in BRAF mutant CRC patients routinely in the clinic, we checked whether these inhibitors alone could provide increased sensitivity to dabrafenib insensitivity in HT-29 cells. From this understanding, collateral drug sensitivity was assessed in dabrafenib-insensitive HT-29 cell population. We initially performed collateral drug testing using oxaliplatin as a second-line therapy in dabrafenib-insensitive HT-29 cells. In this assay, we observed IC50 values of initial, DMSO control, and dabrafenib-insensitive HT-29 cells as 20.25 µM, 28.96 µM, and 7.36 µM respectively. As a result, oxaliplatin showed collateral sensitivity by 74.6% decrease in the IC50 value in dabrafenib-insensitive HT-29 cells in comparison to DMSO control HT-29 cells (Fig. 7A). Next, we checked the effect of capecitabine in initial, control DMSO and dabrafenib-insensitive HT-29 cells using MTT viability assay. Using this assay, we measured the IC50 values for capecitabine sensitivity in initial HT-29, control DMSO, and dabrafenib-insensitive HT-29 cells, and found as 2.8 mM, 3.0 mM, and 1.4 mM, respectively. This finding revealed the presence of collateral sensitivity in dabrafenib-insensitive HT-29 cells against capecitabine as indicated by a decrease of 54.22% in the IC50 value in dabrafenib-insensitive HT-29 cells in comparison to DMSO control HT-29 cells (Fig. 7B). Based on findings where we observed oxaliplatin and capecitabine as inducing collateral sensitivity in dabrafenib-insensitive HT-29 cells, we sought to determine the effect oxaliplatin and capecitabine combination as a second-line treatment in inducing collateral sensitivity in dabrafenib-insensitive HT-29 cells. To do so, we performed MTT cell viability assay using different combination doses of oxaliplatin and capecitabine whereby dabrafenib-insensitive HT-29 cells exhibited increased sensitivity to oxaliplatin and capecitabine combination relative DMSO control and initial HT-29 cell lines (Fig. 7C). Last, we visualized this finding in terms of a change in the dabrafenib sensitivity in different oxaliplatin and capecitabine combination doses of the same experiment and observed significant decrease in the cell viability in dabrafenib-insensitive HT-29 cells in comparison to DMSO control HT-29 cells (Fig. 7D). In our study, we followed the dose range where oxaliplatin and capecitabine showed a sensitivity in dabrafenib-insensitive HT-29 cells using the MTT assays. In the clinic, oxaliplatin is given intravenously for 2 days and cMax concentration is 4.96 µM while capecitabine is given as an oral tablet 2 times a day for the first 14 days where the cMax concentration is 21.1 µM^51^. cMax is defined as the maximum serum concentration of a drug in the patient. As standard of care recommends, these drugs are repeatedly administrated to the patients (for 2 days and 2 times a day) and which may cause an accumulation and leading to increased exposure of a patient to the given drug. Moreover, the cMax concentrations reported as average values and exhibited interpatient variations which can be quite large not only due to patient’s tolerable and metabolic state but also genetic polymorphisms that may be listed as underlying factors^51^. Collectively, these findings indicated the role of capecitabine and oxaliplatin alone and combination as effective second-line therapeutic agents inducing collateral sensitivity in dabrafenib-insensitive HT-29 cells.

## Discussion

Here we show that quantitative measurement of dabrafenib insensitivity in HT-29 cell lines using cellular barcoding technology reveal the presence of distinct barcode distributions, namely pre-existing and de novo barcodes. Longitudinal detection of dabrafenib insensitivity in the HT-29 cell line using harvested used medium allowed for time-dependent and ongoing drug insensitivity within the system. Moreover, whole-exome sequencing analysis revealed possible somatic genomic drivers of dabrafenib insensitivity in HT-29 dabrafenib-insensitive replicate A, presumably associated with dominant pre-existing barcodes detected in this cell line. Furthermore, collateral drug sensitivity testing on dabrafenib-insensitive HT-29 cells revealed that capecitabine and oxaliplatin drugs were effective second-line drugs that increased sensitivity in dabrafenib-insensitive cells. Overall, we provide an in-depth measurement of dabrafenib insensitivity in a cell line model system with an understanding of collateral drug sensitivity.

Our findings were corroborated by the identification of both pre-existing and de novo barcodes, indicating the presence of polyclonal drug insensitivity in HT-29 cell lines exposed to dabrafenib. As a responsible mechanism for drug insensitivity, polyclonal drug insensitivity has previously been reported and is known to associate with intra- and inter-tumor heterogeneity and clonal evolution^52^. CRC encompassing a complex clonal architecture can be measured and monitored with the advent of novel approaches such as the barcoding method used in this study to decipher the underlying molecular dynamics of drug insensitivity^14,28^. Without the power of cellular barcoding approach, common next-generation sequencing-based approaches had limitations to detect rare somatic variants with limited haplotype frequency precision^53^. As demonstrated previously and in this study, a cellular barcoding approach integrated into a model system with a sufficient number of parallel evolutionary replicates allows for the identification of drug insensitivity mechanisms, whether pre-existing/de novo or both^26^. Given the importance of dissecting drug insensitivity mechanisms in cancer therapies, we provide a quantitative understanding for dabrafenib-induced drug insensitivity in a cell line-based model system. However, we would like to acknowledge the limitations of our study as following. The evolutionary dynamics induced by drug treatment may differ between parallel replicates due to stochastic changes in driver alteration behind the insensitivity. The stochastic alterations may even be more prominent in smaller cell populations, such as in our experimental design, compared larger populations (e.g. in Hyperflasks)^26^. Moreover, re-plating applied for each replicate during the course of generation of drug insensitive derivatives in this study may have potentially caused a sampling bottleneck leading to genetic drift and loss of random subclones, which is hard to control experimentally and might explain the observed difference of pre-existing and de novo barcode frequencies between replicates A, B and C. Furthermore, since the data demonstrating cell viability changes for drug insensitivity to dabrafenib from earlier time points than 3 months are not available, the drug insensitivity may well have emerged earlier than 3 months. Tracking the barcode frequency changes through the course of 3 months showed incremental increase in the frequency of single pre-existing barcode (Fig. 4A). Although this data is not based on performing cell viability assays to check drug insensitivity and find out when it is sufficient to draw a conclusion about drug insensitivity in the system, it shows that there is an incremental drug insensitivity gained over the time. Taken together, we acknowledge them as limitations of our study.

It was previously reported that developing insensitivity to BRAF inhibitors in BRAF-mutant colorectal cancer patients may take around few months^54–57^. Based on this information, we interpreted the duration for dabrafenib exposure to generate insensitive derivatives of BRAF mutant HT-29 cells as 3 months, also partly because of the experience of our laboratory for generating insensitive cell lines take several months. In the current standard of care, dabrafenib is recommended as 150 mg, 2 times a day for a patient^58^. Moreover, dabrafenib used in the clinic comes as a tablet, and it is hard to reach a conclusion about its effective concentration at cancerous site in patients. For these limitations, we first identified IC50 concentration of dabrafenib using initial HT-29 cell line and based on this concentration, we designed our in vitro experiment to expose these cells to 2XIC50 (199.6 nM) dabrafenib to generate their insensitive derivatives. Despite cell lines were considered as useful model systems, they may exhibit limitations in mimicking drug response seen treatment of cancer patients. We acknowledge this as the limitation of our current study. Furthermore, cMax concentration recommended to be used in patients may not reflect in vitro setting as patients are treated with a repeated dosing schedule which makes it harder to recapitulate. Moreover, recommended cMax doses may exhibit interpatient variation depending on patient’s tolerable, metabolic states in addition to genetic polymorphisms.

In this study, incorporating barcode sequences into the initial HT-29 cells allowed for the monitoring of evolutionary insensitivity for ongoing experiments via medium harvesting. As a result, we were able to monitor temporal drug insensitivity in a time-dependent manner, allowing quantitative measurement of clonal evolution in HT-29 cells under the pressure of dabrafenib. This novel approach utilized previously only once by us in a different cancer setting provided a powerful look into ongoing dabrafenib insensitivity in HT-29 cells with the help of multiple data points rather than single end point. This in fact shows a similarity with liquid biopsy approaches where tracking of mutations in cancer genes provided in depth understanding for longitudinal clonal evolution^59^. In this study, we found a list of SNVs and CNVs in dabrafenib insensitive HT-29 cells in comparison to DMSO HT-29 cells. Of note, these genomic changes might be attributed to early resistance stage of HT-29 under dabrafenib exposure since the length of dabrafenib treatment in our system was 3 months. Longer than 3 months of dabrafenib exposure might be required to observe full dabrafenib resistance. The transition from pre-resistant stage to stably resistant stage requires prolonged exposure of treatment and monitoring the acquisition of additional heritable epi(genetic) markers within system. While the pre-resistant stage confer survival for a subpopulation(s) under the drug exposure, acquiring additional secondary genomic alterations provide full resistant stage, preventing to revert drug-sensitive stage. Since dabrafenib-insensitive HT-29 replicate A was dominated by pre-existing barcodes (in Fig. 3, frequency measurement releveled that 99.3% was pre-existing), according to barcode analysis, majority of the genomic changes detected by the WES analysis might belong to the pre-existing barcodes. This being said, we cannot rule out the contribution of remaining minor de novo barcodes in mediating the drug insensitivity in our system. To explain possible involvement of pre-existing barcodes in replicate A in dabrafenib insensitivity, we suggest that CNV gain in *SGK1* gene found in our study might govern the insensitivity associated with pre-existing barcodes as *SGK1* was previously reported as downstream of YAP1 transcriptional activator activating ERK1/2 signalling as a possible mechanism of BRAF V600E insensitivity in CRC cells^37^. We would like to highlight this as only suggestive and future experiments will be needed to elucidate it. These experiments will include (1) the establishment of single-cell derived cell lines harbouring a pre-existing or a de novo barcode followed by identifying their genomic architecture and response to second-line drugs. (2) expressible barcode system integrated with scRNAseq to find out the expression profiles clustered subclones with pre-existing or de novo barcodes before and after second-line treatments. Being unable to show single-cell dynamics of minor pre-existing and de novo subpopulations that might mediate dabrafenib-insensitivity with the use of listed future experiments, we would like to acknowledge them as limitations of our study.

Of note, 99.3% dabrafenib-insensitive HT-29 replicate A population is consisted of a total of four pre-existing barcodes forming the pre-existing subpopulations. Among these four pre-existing barcodes, one of them, coloured with blue in Fig. 4A, exhibited an increased frequency over the duration for insensitivity generation and overlapped with final harvested population as assessed by the temporal barcode tracking experiment. The frequency of this barcode was found as 99.05% at final time point, indicating that dabrafenib insensitivity in this cell line was dominated by this subpopulation. To characterize the contribution of subpopulations to drug insensitivity, we indeed performed WES and our analysis identified number of SNVs and CNVs that could be attributed to this major clone due to its 99.05% frequency in whole population. We acknowledge the presence of remaining pre-existing (0.25%) and de novo (0.37%) subpopulations since they may contribute to drug insensitivity in our system; however due to their very low frequencies, we argue that their contribution would be very minor. As it was commonly recommended, bioinformatic analysis of WES data for the identification of variant allele frequencies was based on detection limit of minimum allele frequency of 0.1. With the current depth of WES in our study (202X), it would not be possible to detect variant allele frequencies less than 10% for the minor subpopulations. Moreover, characterization of minor drug insensitive subpopulations would require performing WES analysis with at least 10 times higher sequencing depth than being used in this study. Given the inability to characterize genomic profiling of minor subpopulations, we would like to acknowledge them as limitation of our study.

Developing insensitivity to targeted therapy can open a window of opportunity for a second-line therapies sensitizing initially drug insensitive populations, a phenomenon known as collateral drug sensitivity^30,31,48^. As we previously demonstrated in non-small cell lung cancer model system^26^, this time here in this study, we show that capecitabine and oxaliplatin induce collateral sensitivity in dabrafenib-insensitive HT-29 cell lines. Although capecitabine and oxaliplatin are frequently used as adjuvant therapies in the treatment of CRC patients, their efficacy as second-line therapeutics is unknown^60^. In this study, dabrafenib insensitivity developed polyclonal drug insensitivity, as determined by barcode sequencing, resulting in a change in clonal composition and, as a result, a change in sensitivity to capecitabine and oxaliplatin compared to the initial and DMSO control HT-29 cell lines. Because of the nature of intratumor heterogeneity, where cancer is formed by multiple genetically and phenotypically distinct subpopulations, it is more difficult to overcome insensitivity and target multiple pre-existing and de novo insensitivity^14,45^. However, based on the understanding of evolutionary therapy^44,61^, we found that dabrafenib induced a change in the clonal landscape in HT-29 cells, and thus the sensitivity.

## Materials and methods

### Cell culture

HT-29 cells were purchased from Şap Institute, Ankara, Turkey where the quality and identify were validated. HT-29 cells were cultured using High Glucose (4.5 g/l) Dulbecco’s Modified Eagle Medium (DMEM) (Biological Industries, Israel), 10% (v/v) Fetal Bovine Serum (FBS) (Biological Industries, Israel), 1% (v/v) Penicillin–Streptomycin (Biological Industries, Israel) and 1% (v/v) l-Glutamine (Biological Industries, Israel). HEK293T cells were obtained from the American Tissue Culture Collection and cultured using High Glucose (4.5 g/l) DMEM, 10% (v/v) FBS (Biological Industries, Israel), 1% (v/v) Penicillin–Streptomycin (Biological Industries, Israel). In total of 3 months for generation of dabrafenib-insensitive replicates A, B and C, replicate A was passaged 4 times, while replicates B and C were passaged 3 times. In each passaging, 10% of total cell number were re-seeded to continue the experiment while remaining 90% of total cell number were discarded. To confirm the mycoplasma negativity, PCR-based method was used.

### Barcoding of the cell line

Barcoding of HT-29 cell line was achieved using CloneTracker™ lentiviral barcode library (Cellecta, USA). HEK293T cell line was used for the generation of lentiviruses transfected with the following plasmids: barcode library, pCMV-VSVG (Addgene: 8454), and Delta 874.LV (Addgene:8455). The infection of HT-29 cells with lentivirus was carried out using 0.8 μg/ml polybrene. Lentiviral titration was performed to achieve Multiplicity of infection (M.O.I.) 0.05, and 1.5 μg/ml puromycin concentration was used for the selection of barcode library in HT-29 cells.

### Establishment of dabrafenib-insensitive HT-29 cell line

Barcoded HT-29 cells frozen and thawed for the establishment of dabrafenib-insensitive counterparts. A total of 5 × 10^6^ barcoded HT-29 cells were thawed and seeded into a 15 cm tissue culture dish. Following the cells reaching the confluency, they were trypsinized and well-mixed before seeding equal number of cells (2 × 10^6^ cells) per new 15 cm dishes. A total of 4 dishes that are namely DMSO Control, Replica A, B, and C was formed. As an initial time-point, frozen cell stocks and cell pellets from 2 × 10^6^ cells in each were collected. Harvesting used medium through the experiment was performed at monthly intervals. Barcoded HT-29 cell line replicates A, B, and C were treated with 2XIC50 (199.6 nM) of dabrafenib concentration for the duration of 3 months.

### Barcode analysis using amplicon next-generation sequencing

Both end points after 3 months of dabrafenib treatment and harvesting of used medium samples were centrifuged at 1200 rpm to generate pellets. Pellets were then processed for genomic DNA (gDNA) isolation using GeneJET Genomic DNA isolation kit (ThermoFisher, USA). For amplicon NGS library preparation, Cellecta’s manufacturer guidelines were followed whereby amplification of barcode sequences was carried out via amplicon PCR using forward and reverse primer sequences. Finally, 14 bp and 30 bp length variable barcode sequences were sequenced on MiSeq Platform (Illumina, Inc.) using 150 bp pair-end method.

### Barcode frequency analysis

Following the FASTQ dataset were obtained from the Illumina MiSeq platform, FASTQC was used to assess base qualities whereby Phred scores less than 20 were excluded. FASTQ files were then trimmed using the trimmomatic with a following criteria to eliminate pair-end reads less than 147 bp. Next, Cellecta barcode library, provided by the manufacturer, was used to generate 1 million barcode sequences. Finally, detected barcode reads was written in SF file using the Salmon function and a CSV file conversion was carried out to identify barcode counts. This was then followed by the identification of unique barcode counts based on their frequencies in dabrafenib insensitive replicates A, B and C relative to DMSO control HT-29 cells. Last, our previous formula to calculate growth rates were used to find barcode phenotypes^26^:

$$r=\frac{1}{T}log \left(\frac{{f}_{R}}{{f}_{0}} \right)$$

In the formula, frequency of barcodes in the replicate was assigned to f_R_ and number of barcodes within the DMSO control group was assigned to f_0_, while T shows the time in weeks between the drug applied and end of the experiment. In the analysis, barcode counts with less than 2 in all insensitive replicates were excluded. Barcodes with positive growth rates derived from the formula and detected at least in 2 replicates were named as “pre-existing”, while with positive growth rate and detected only in replicate were named as “de novo”. Remaining barcodes with negative growth rate were named as “sensitive”.

### Harvesting floating barcodes from used medium

The used medium was replaced with fresh one twice a week containing 2XIC50 (199.6 nM) dabrafenib concentration for the duration of 3 months. Floating DNA harboring barcode sequences were centrifuged and collected as pellets before the gDNA isolation. gDNAs representing equal time intervals for the duration of experiment were faced to barcode amplicon NGS analysis.

### Whole exome sequencing

Whole exome sequencing libraries of DMSO control and dabrafenib-insensitive A cell lines were prepared from genomic DNA by using Twist Comprehensive Exome kit according to the manufacturer’s instructions. Following pooling of libraries, samples were sequenced on the MGI DNBSEQ-G400 platform. The mean coverage was determined by Qualimap tool as 229X for DMSO and 202X for the insensitive line. Quality control of fastq files revealed that the quality parameters were good enough so there was no need for further pre-processing. Reads were aligned to hg38 reference genome by using the Burrows–Wheeler Aligner tool. GATK (v4.3.0.0) Mutect2 was used to call somatic variants after duplicate marking and base recalibration. Variants were annotated by Funcotator and filtered in a way that only the non-synonymous variants that have a minimum alternative coverage of 10 reads, minimum allele frequency of 0.1 and a location within the target regions of the exome capture panel were kept for further analysis (Supplementary Tables 3 and 4). Variants exhibiting at least 2 × enrichment/depletion in VAF in replicate A line compared to DMSO control were determined (Supplementary Table 5). Control-FREEC (v.11.6)^62^ tool was used to detect copy number alterations (CNA) using DMSO line as a control and assuming a ploidy 3. Significant CNAs (p-value < 0.05) were identified (Supplementary Table 6).

### Dose response analysis

Dabrafenib-insensitive, DMSO control and initial HT-29 cells were seeded into 96-well plates at 10 × 10^3^ cells/well density. After 24 h of seeding, a medium was replaced with drugs according to dose range. The drugs were used as following: dabrafenib (AdooQ Bioscience, USA), oxaliplatin (LC Laboratories, USA.), capecitabine (LC Laboratories, USA). After 72 h of drug treatment, MTT cell viability assay was performed. We used GraphPad Prism software to calculate IC50 values in all cell viability results through following the software’s guidelines^63^. The drug concentrations used in the experiment were initially transformed as a logarithmic concentration. Then, we analysed the data using log(inhibitor) vs. normalised response model and non-linear regression. The dose response model estimated the IC50 value according to the rest of data points used in the regression model.

### Growth rate analysis

Dabrafenib-insensitive HT-29, DMSO controls, and initial cell populations were seeded into separate 96 well plates at 10 × 10^3^ cells per well. After 24 h, DMSO control HT-29 cells and dabrafenib-insensitive HT-29 cells were treated with DMSO and dabrafenib, respectively, while the initial control cells were given full fresh growth medium. After 24 h, 48 h, and 72 h in the incubator, the cells were fixed, stained with crystal violet and destained with 20% acetic acid. Finally, absorbance values were measured at 570 nm using a microplate spectrophotometer (Multiskan GO; Thermo Fisher Scientific, USA). Cell proliferation rates were calculated using GraphPad Prism 8 and absorbance values (GraphPad Software Inc., USA).

### Colony formation assay

In 6-well plates, 20.000 cells were seeded per well and allowed to attach for 24 h. The treatments began with 2XIC50 dabrafenib and DMSO, and the medium was changed twice a week. After thirty days, the cells were fixed with a 2% formaldehyde (Serva, USA) solution diluted in phosphate-buffered saline (PBS) (Biological Industries, Israel). After 2 h, the cells were stained in water with 0.1% crystal violet (Amresco, USA). Eight minutes later, the staining solution was washed away with dH_2_O and allowed to dry overnight.

### Scratch assay

The scratch assay was used to examine the migration of drug-insensitive, DMSO-control, and dabrafenib-insensitive HT-29 cell lines. A 24-well plate containing growth was seeded with an equal number of cells per cell line group (4 × 10^5^/well). After the cells had reached approximately 80% confluence, they were treated for 2 h with 2 g/ml Mitomycin (Serva, VWR International). Scratches were created with a 200 μl pipette tip, and the cells were washed three times to remove debris. DMSO control HT-29 cells were treated with DMSO, while dabrafenib-insensitive HT-29 cells with dabrafenib and fresh growth medium was given to the initial control cells. Cell migration was monitored every 4 h using a Nikon Eclipse Ti2e microscope. The area of closure was calculated via ImageJ.

### Statistical analysis

Each experiment had at least three biological replicates and three technical replicates. The dose–response curve data was analyzed using GraphPad Prism 8. (GraphPad Software Inc., USA). The Student's *t*-test, two-way ANOVA, and nonlinear regression were used to determine statistical significance. Statistical significance was defined as p-values less than 0.05. All results were presented as mean SEM.

### Supplementary Information

Supplementary Information.

Supplementary Table 3.

Supplementary Table 4.

Supplementary Table 5.

Supplementary Table 6.

## Data Availability

Barcode sequencing and whole-exome sequencing data generated in this study were deposited into ArrayExpress-The functional genomics data collection with the accession numbers of E-MTAB-13018 (https://www.ebi.ac.uk/biostudies/arrayexpress/studies/E-MTAB-13018) and E-MTAB-13338 (https://www.ebi.ac.uk/biostudies/arrayexpress/studies/E-MTAB-13338), respectively.
