# Supplementary material for: Characterization of dabrafenib-induced drug insensitivity via cellular barcoding and collateral sensitivity to second-line therapeutics
Source: Sci Rep. 2024 Jan 2;14:286. doi: 10.1038/s41598-023-50443-3 (PMC10762103; doi:10.1038/s41598-023-50443-3)
Supplement: Supplementary file 1 — Supplementary Information. [file 41598_2023_50443_MOESM1_ESM.docx]

**Characterization of dabrafenib-induced drug insensitivity via cellular barcoding and collateral sensitivity to second-line therapeutics**

**Rana Can Baygin^1^, Kubra Yilmaz^1^, Ahmet Acar^1, *^**

**^1^** Department of Biological Sciences, Middle East Technical University,

Universiteler Mah. Dumlupınar Bulvarı 1, 06800 Çankaya, Ankara, Turkey

* To whom correspondence should be addressed: [acara@metu.edu.tr](mailto:acara@metu.edu.tr)


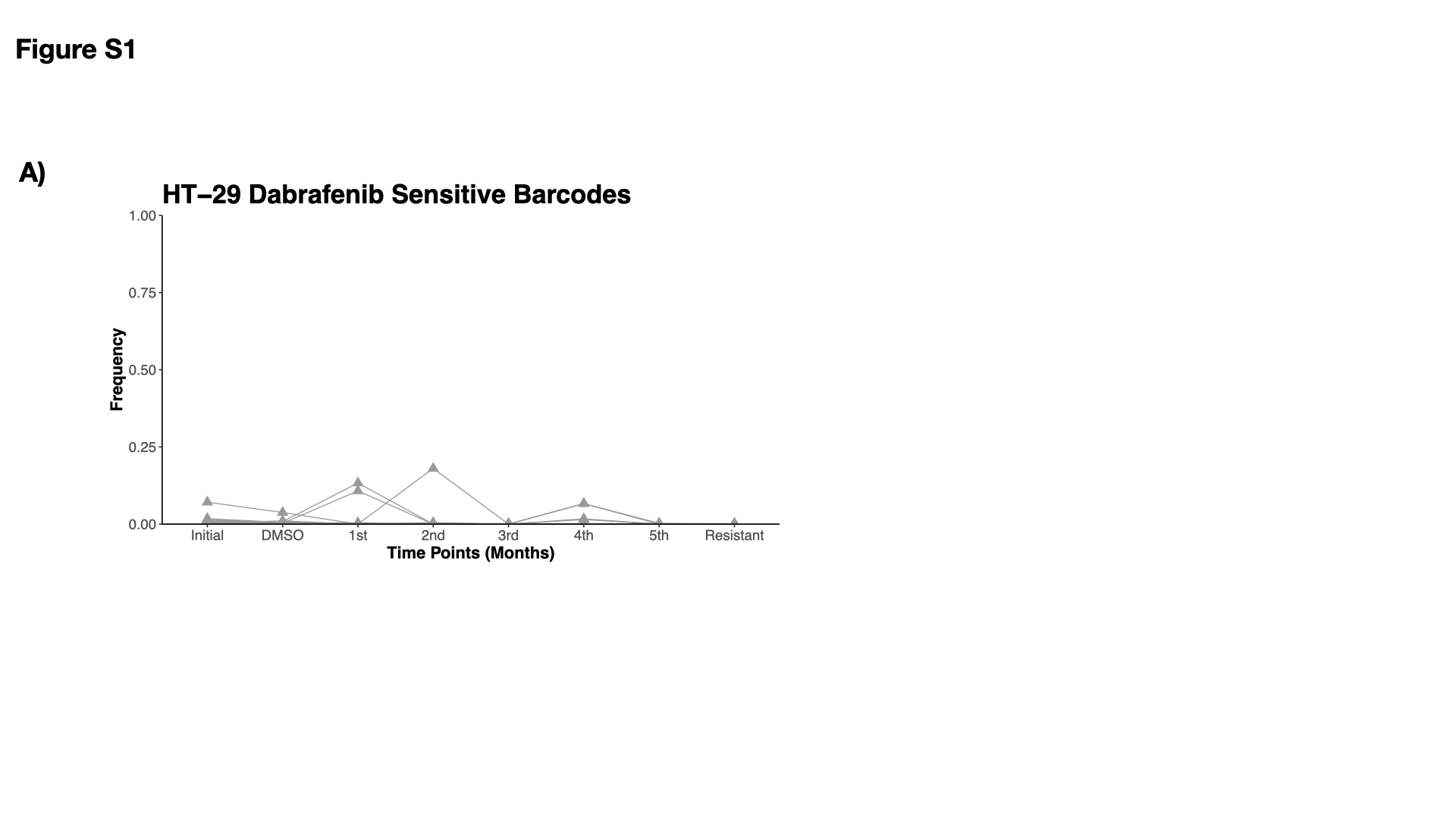


**Supplementary Figure 1. Barcode frequency measurements of the sensitive barcodes. A.** Frequencies of sensitive barcodes in dabrafenib-insensitive HT-29 cell line replicate A was shown.


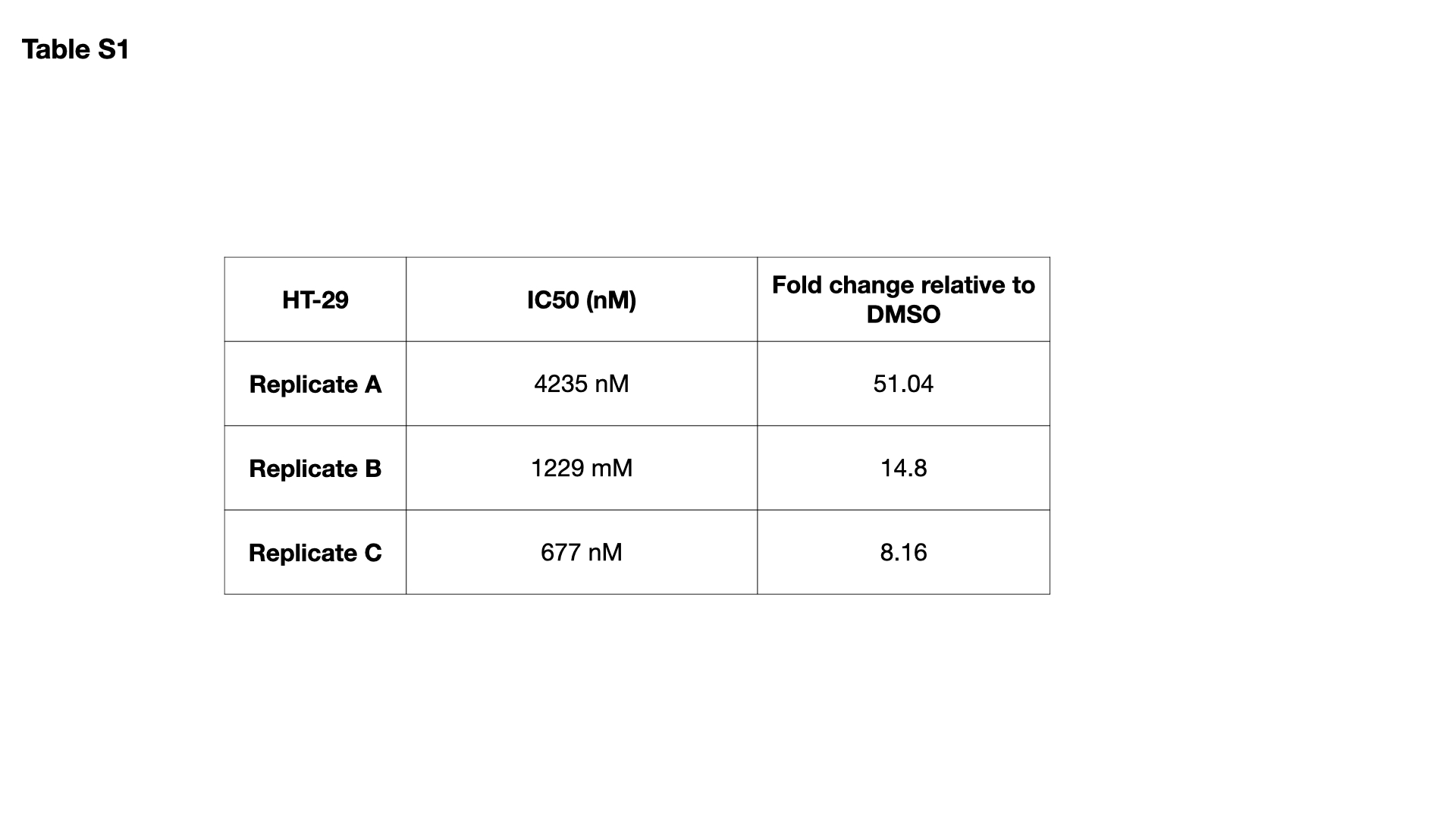


**Supplementary Table 1.** Fold change calculations in dabrafenib- insensitive HT-29 cell lines replicates A, B, and C in comparison to DMSO control HT-29 cell line were demonstrated.


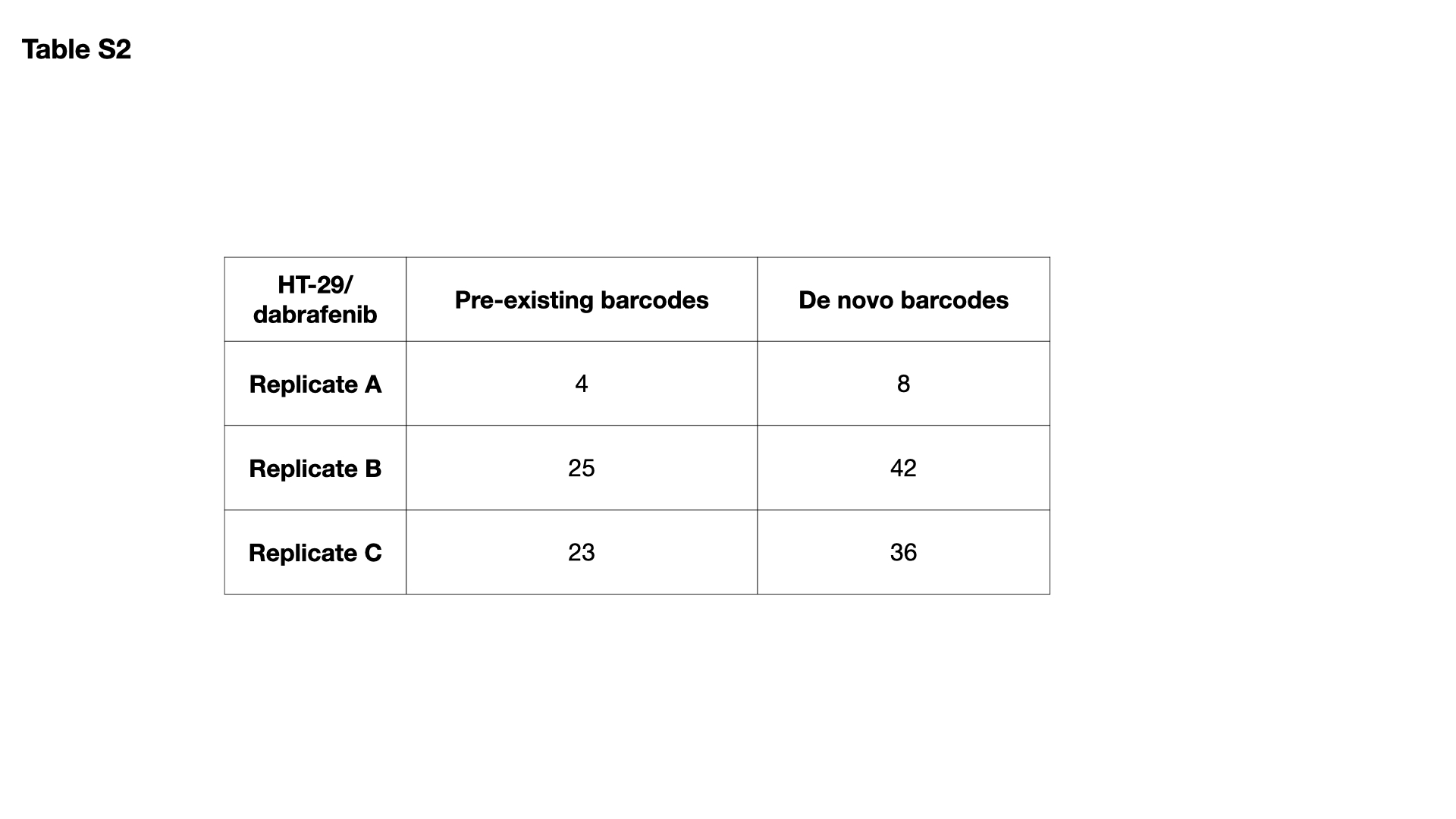


**Supplementary Table 2.** Numbers for pre-existing and de novo barcodes in dabrafenib-insensitive HT-29 cell line replicates A, B, and C.

**Supplementary Table 3. Whole-exome sequencing analysis DMSO HT-29 filtered variants**.

**Supplementary Table 4. Whole-exome sequencing analysis Dabrafenib-insensitive HT-29 replicate A filtered variants**.

**Supplementary Table 5. Whole-exome sequencing analysis SNV comparison results.**

**Supplementary Table 6. Whole-exome sequencing analysis CNV comparison results.**
